# Hypophosphatemic Osteomalacia in a Young Adult

**DOI:** 10.7759/cureus.13697

**Published:** 2021-03-04

**Authors:** Firjeeth Paramba, Manju Silas, Naseer Masoodi, Silas Benjamin, Jafer Ajanur Palaki

**Affiliations:** 1 Internal Medicine, Hamad Medical Corporation, Doha, QAT; 2 Family Medicine, Al Wakra Primary Health Centre, Doha, QAT

**Keywords:** hypophospotemic osteomalacia, tumor induced osteomalacia, fgf

## Abstract

Tumor-induced osteomalacia (TIO), otherwise known as oncogenic osteomalacia, is a rare paraneoplastic syndrome, characterized by hypophosphatemia due to decreased tubular reabsorption and low or inappropriately normal level of active vitamin D. The syndrome, first recognized by Robert McCance in 1947, is well described in the medical literature. However, the diagnosis can be delayed due to the nonspecific nature of its presentation. The tumor responsible for TIO produces fibroblast growth factor 23 (FGF-23) which plays a role in regulating renal handling of phosphate and 25-hydroxyvitamin D 1α-hydroxylase activity. Chronic hypophosphatemia eventually leads to inadequate bone mineralization and osteomalacia. The diagnosis should be considered when a patient presents with low phosphate and osteomalacia or rickets and should be differentiated from other disorders of phosphate metabolism such as X-linked, autosomal dominant and recessive hypophosphatemic rickets, and acquired cause like vitamin D deficiency. The localization of the tumor is rather difficult as the tumor can be too small and be anywhere in the body. A combination of thorough physical examination, laboratory tests, and proper imaging is needed for the diagnosis. Surgical removal of the tumor often leads to complete resolution of the syndrome. If the tumor is undetectable or unresectable, then phosphate and vitamin D supplements should be considered.

## Introduction

Tumor-induced osteomalacia (TIO) is characterized by bone pain, muscle weakness, and fractures associated with persistent hypophosphatemia due to renal phosphate wasting, inappropriately normal or low 1,25 dihydroxy vitamin D, and elevated fibroblast growth factor 23 (FGF-23). TIO is caused by tumoral overproduction of FGF-23 that acts primarily at the proximal renal tubule to inhibit phosphate reabsorption and 1α-hydroxylation of 25-hydroxyvitamin D which leads to hypophosphatemia and subsequent osteomalacia.

## Case presentation

A 34-year-old male with no significant past medical history presented with generalized body pain and swelling of multiple joints. His symptoms slowly progressed and he developed severe backache and body pain which forced him to walk with support. Pain was so severe that someone had to help him get into his car. He had no history of trauma and was not on any medication. He did not have fever or other systemic symptoms. Initial clinical examinations were normal apart from generalized bone tenderness. The systemic examination did not reveal any features of Cushing’s syndrome.

The laboratory investigations showed elevated alkaline phosphatase, low serum phosphate, normal levels of parathyroid hormones (PTH), calcium, and vitamin D (Table 1). His routine blood count, renal function, and serum protein electrophoresis were normal. The urine test was negative for Bence Jones protein.

**Table 1:** Laboratory findings

Tests	Result
Serum calcium	2.21 mmol/L (2.10-2.55)
Phosphate	0.49 mmol/L (0.74-1.52)
Alkaline Phosphate	338 u/L (40-150)
Vitamin D	35 ng/mL
PTH (Parathyroid Hormone)	28 pg/ml (8.0-74)
Tubular Maximum Reabsorption Phosphate/Glomerular Filtration Rate	1.9mg/100 ml (Age based normal TmP/GFR in males (2.5-3.5 mg/dl)

**Figure 1:**
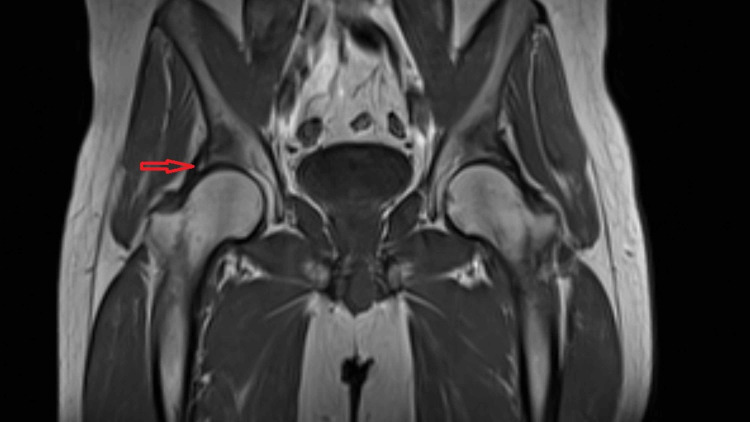
Coronal slice of T1-weighted MRI showing fracture of acetabulum (red arrow)

**Figure 2:**
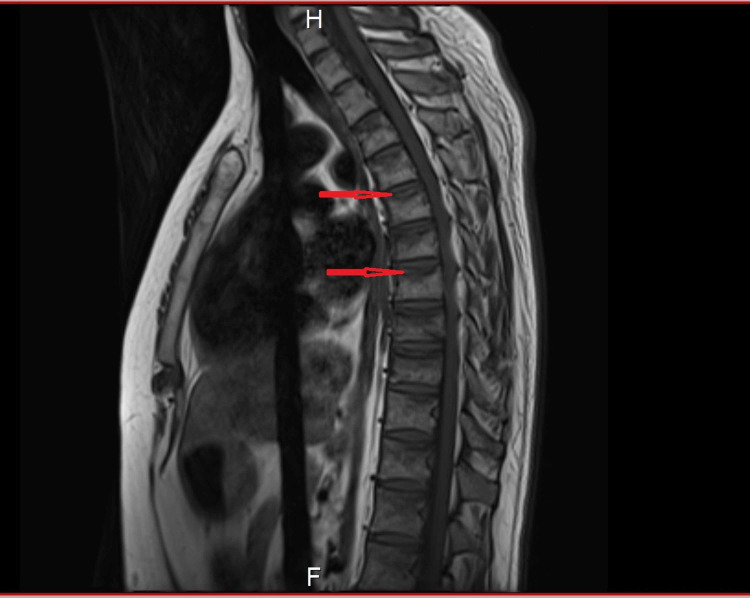
Sagittal slice of a T1-weighted MRI showing multiple vertebral compression fractures along the superior end plates (red arrows)

**Figure 3:**
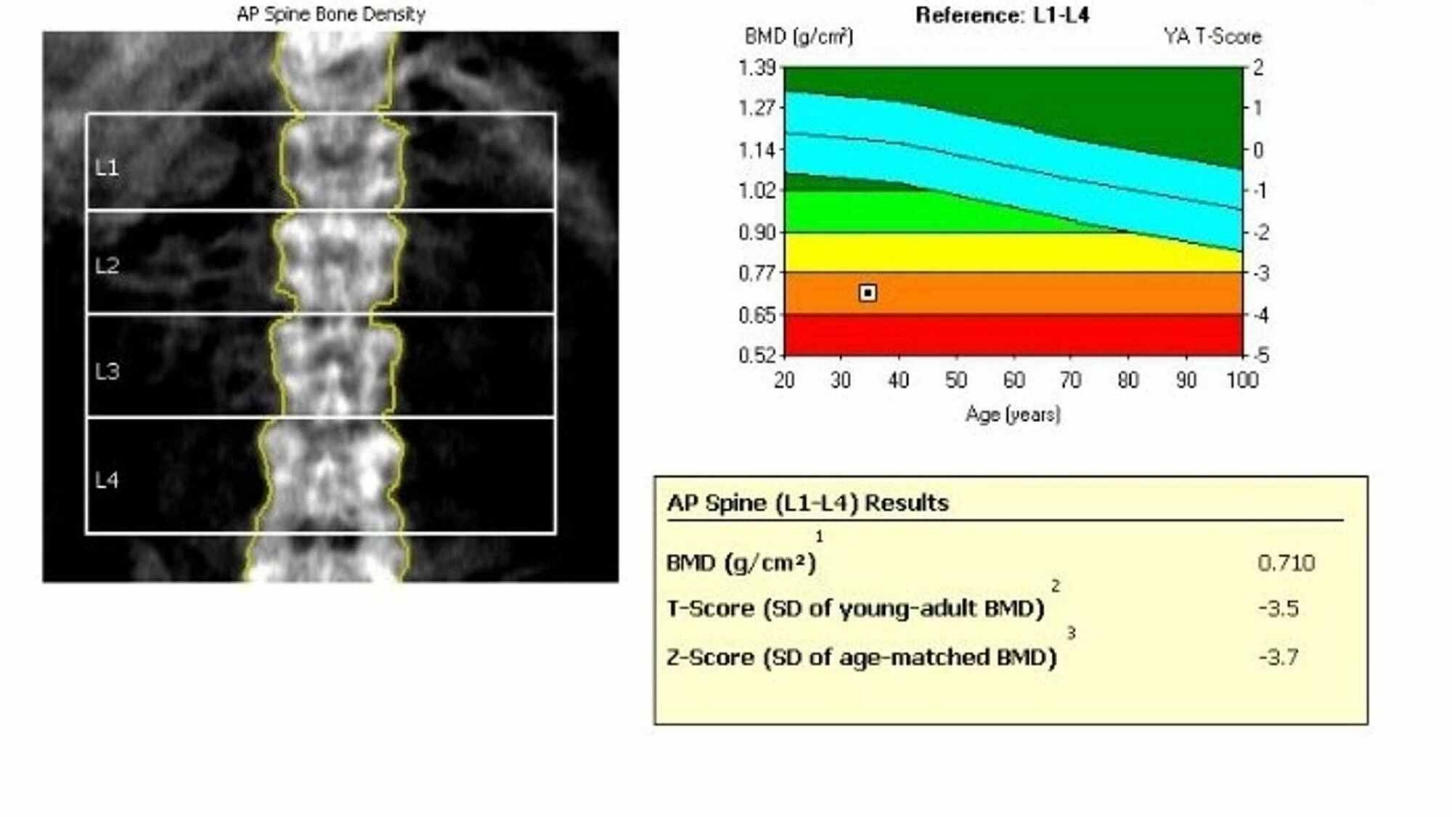
Bone mineral density (BMD)

X-ray of the cervical, thoracic and lumbar spine, shoulder, pelvis, and sacroiliac joint were also normal. Due to multiple joint pain and bone tenderness, a whole body MRI scan was done which was suggestive of osteoporosis with multiple insufficiency fractures of the sacrum, distal end of tibia and fibula, ribs, and acetabulum as well as multiple compression fractures of the vertebral body (Figures 1-2). The bone densitometry showed a T score of -3.5 in the lumbar spine (Figure 3). 

**Figure 4:**
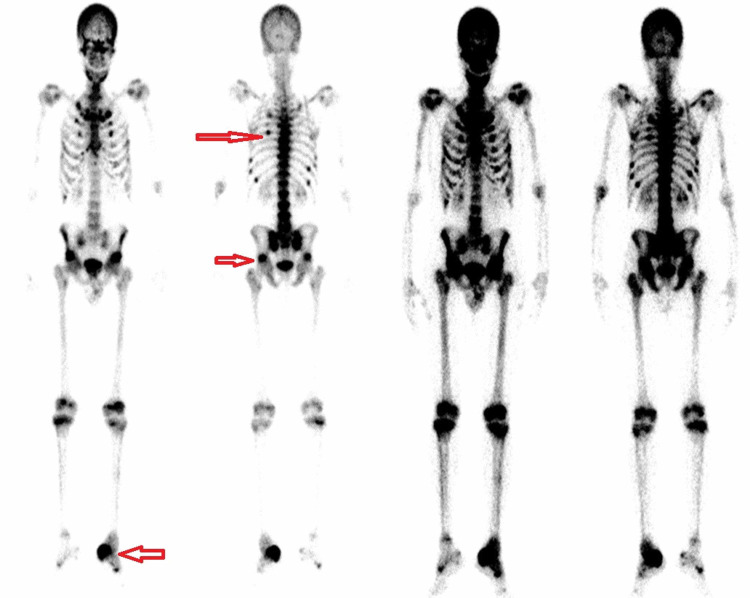
Dual-phase bone scan showing increased radiotracer uptake at costochondral junctions, bilateral acetabulum, and left calcaneum (red arrows)

A subsequent bone scan revealed multiple foci of increased radiotracer uptake likely from Looser's zones/pseudo fractures and costochondral beadings. Also increased uptake at proximal femoral and tibial growth plates likely from pseudo reactivation of growth plates. Multiple levels of increased vertebral uptake were noticed from compression fractures and at the sacroiliac joints from sacral insufficiency fracture. Findings were highly suggestive of osteomalacia. Left calcaneus three-phase bone scan revealed positive uptake suggestive of insufficiency type stress fracture. Delayed bone phase also showed bilateral distal tarsal/proximal metatarsal uptakes indicative of pseudo fractures/stress fractures (Figure 4).

He was then reviewed at an endocrine clinic and further work up was done for hypophosphatemia and metabolic bone disease. The tubular maximum reabsorption of phosphate to glomerular ﬁltration rate ratio (TmP-GFR) was 1.9 mg/100 ml (2.5-3.5 mg/dl) suggestive of TIO. 

**Figure 5:**
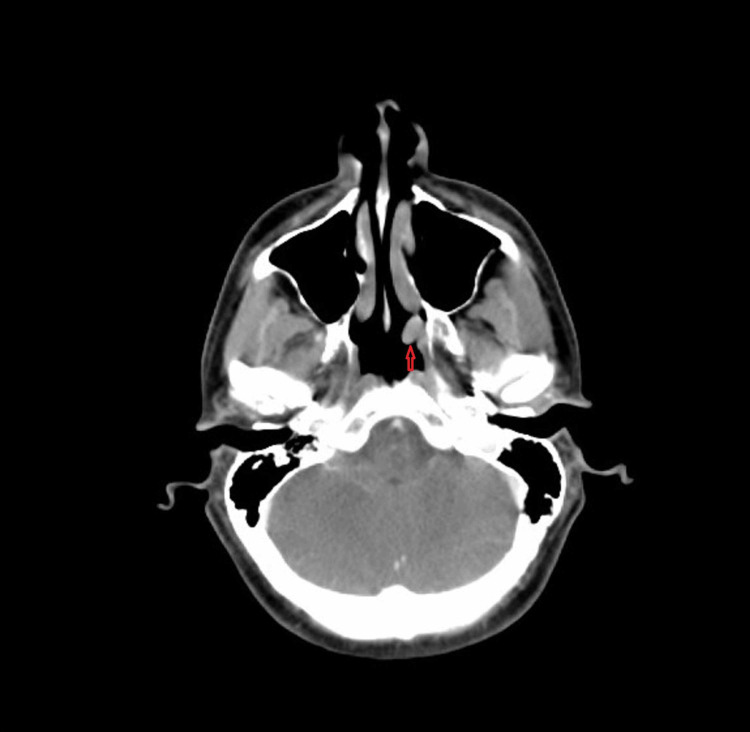
CT image showing soft tissue lesion located at the lateral wall of the left nasal cavity (red arrow)

**Figure 6:**
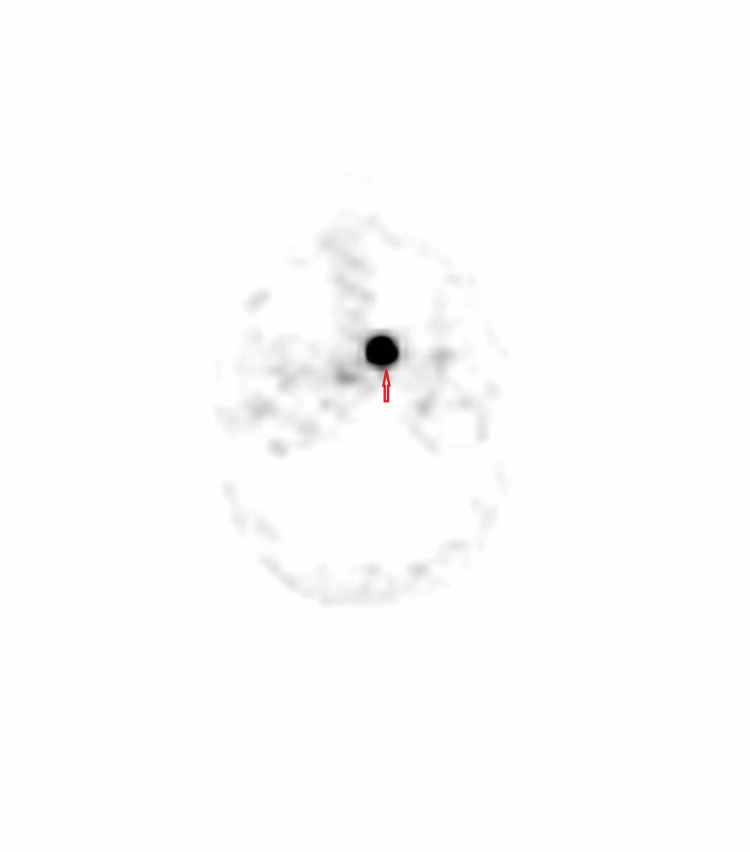
Dotatate positron emission tomography (PET)/CT scan showing intense uptake at choanal lesion

In order to locate the tumor, a whole body CT scan was done which detected a vascular lesion arising from the lateral wall of the left nasal cavity at the level of the choana (Figure 5). A subsequent nasopharyngoscopy revealed a vascular tumor at the posterior end of the inferior turbinate and the positron emission tomography (PET) scan showed uptake in the same region (Figure 6).

He underwent excision of the tumor and histology confirmed a hemangiopericytoma. Patient had a marked clinical and biochemical improvement on follow up and started walking independently within few weeks. His repeat bone mineral density after two years showed a T score of -1.1 in the lumbar spine.

## Discussion

TIO, though well recognized in literature [1], has an unknown prevalence and is easily missed in clinical practice [2]. Histologically, 70%-80% of mesenchymal tumors associated with TIO are classified as phosphaturic mesenchymal tumors/mixed connective tissue tumors (PMT/MCT) which include hemangiopericytomas [3]. These tumors are usually slow growing, benign and small. They are commonly found in the craniofacial region or in the extremities [4]. Other histological types include fibromas, chondrosarcomas, neuroblastomas, and prostate carcinomas. Locating the tumor is a critical step in the management of TIO because surgical resection of the tumor can often lead to a complete resolution of the syndrome. 

The biochemical hallmark of the disorder is hypophosphatemia due to inadequate renal phosphate absorption. Other causes of hypophosphatemia include intestinal malabsorption, intercellular shifts or defect in renal reabsorption of phosphate. In a case of hypophosphatemia, renal wasting of phosphate should be suspected if serum calcium, vitamin D and PTH levels are normal and other systemic illnesses are ruled out. A 24-hour urine collection for phosphate will be high in hypophosphatemia due to increased renal loss.

FGF-23 encoded on FGF-23 gene located on chromosome 12, produced by osteocytes is responsible for pathophysiological changes in TIO. The FGF-23 reduces serum 1,25 dihydroxy vitamin D level and suppresses renal reabsorption of phosphate resulting in hypophosphatemia and metabolic bone disease.

The elevated serum FGF-23 is a useful marker for diagnosis and follow up evaluation of TIO. Most cases of TIO have elevated levels of FGF-23 which causes inadequate homeostatic response leading to hypophosphatemia and defective vitamin D synthesis. FGF-23 has a short half-life which makes it a useful tumor marker and can be measured intraoperative as well as immediately after operation to assess the effectiveness of the surgery [5,6].

The genetic conditions like X-linked hypophosphatemic rickets, autosomal dominant rickets and fibrous dysplasia caused by mutations to PHEX (phosphate regulating endopeptidase homolog X-linked) gene, can also present with elevated levels of FGF-23 and cause renal phosphate wasting. However, TIO usually occurs in adulthood.

The calculation of the ratio of TmP/GFR is an alternate way to assess phosphate homeostasis. In TIO-associated hypophosphatemia, the TmP/GFR is lower than expected for a given serum phosphorus concentration [7,8].

After the biochemical confirmation, localizing the tumor is crucial. The routine CT or MRI may miss the site of tumor and hence a functional imaging using 68 Ga Dotatate PET/CT with octreotide or 18F-fluorodeoxyglucose (18F-FDG) PET/CT is recommended. All functional imaging should include entire body. The studies have shown that 68 Ga Dotatate PET/CT has higher sensitivity and specificity in localizing tumor compared to other functional imaging [9].

In case of multiple lesions, selective venous sampling with FGF-23 measurement will help in localizing them. If the tumor is still not identified, then a periodic follow up and further imaging at a later stage is recommended. Once identified, the removal of the tumor is recommended though it may be challenging in case of head and neck cancers [10]. A complete resection of the tumor leads to rapid normalization of serum phosphate and FGF-23 levels.

If the tumor is inaccessible or inoperable, then a regular follow up is to be arranged in order to avoid complications such as secondary or tertiary hyperparathyroidism. Medical therapy with phosphate supplements, calcitriol or alfacalcidol should be considered in those cases. Newer treatments like image guided ablation, monoclonal antibodies, and anti fibroblast growth factor receptor (FGFR) medications can be recommended for the management of unresectable tumors causing oncogenic osteomalacia [11].

## Conclusions

TIO is not an uncommon disorder. Patients presenting with nonspecific musculoskeletal symptoms with biochemical evidence of hypophosphatemia should be evaluated for TIO. Localisation of the lesion is challenging in some cases and sometimes warrant functional imaging and regular follow up.
